# Broadly sampled assessment reduces ethnicity‐related differences in clinical grades

**DOI:** 10.1111/medu.13790

**Published:** 2019-01-25

**Authors:** Chantal E E van Andel, Marise Ph Born, Axel P N Themmen, Karen M Stegers‐Jager

**Affiliations:** ^1^ Institute of Medical Education Research Rotterdam Erasmus MC Rotterdam the Netherlands; ^2^ Department of Psychology Erasmus University Rotterdam Rotterdam the Netherlands; ^3^ Department of Internal Medicine Erasmus University Rotterdam Rotterdam the Netherlands

## Abstract

**Context:**

Ethnicity‐related differences in clinical grades exist. Broad sampling in assessment of clinical competencies involves multiple assessments used by multiple assessors across multiple moments. Broad sampling in assessment potentially reduces irrelevant variances and may therefore mitigate ethnic disparities in clinical grades.

**Objectives:**

Research question 1 (RQ1): to assess whether the relationship between students’ ethnicity and clinical grades is weaker in a broadly sampled versus a global assessment. Research question 2 (RQ2): to assess whether larger ethnicity‐related differences in grades occur when supervisors are given the opportunity to deviate from the broadly sampled assessment score.

**Methods:**

Students’ ethnicity was classified as Turkish/Moroccan/African, Surinamese/Antillean, Asian, Western, and native Dutch. RQ1: 1667 students (74.3% native Dutch students) were included, who entered medical school between 2002 and 2004 (global assessment, 818 students) and between 2008 and 2010 (broadly sampled assessment, 849 students). The main outcome measure was whether or not students received ≥3 times a grade of 8 or higher on a scale from 1 to 10 in five clerkships. RQ2: 849 students (72.4% native Dutch students) were included, who were assessed by broad sampling. The main outcome measure was the number of grade points by which supervisors had deviated from broadly sampled scores. Both analyses were adjusted for gender, age, (im)migration status and average bachelor grade.

**Results:**

Research question 1: ethnicity‐related differences in clinical grades were smaller in broadly sampled than in global assessment, and this was also seen after adjustments. More specifically, native Dutch students had reduced probabilities (0.87–0.65) in broadly sampled as compared with global assessment, whereas Surinamese (0.03–0.51) and Asian students (0.21–0.30) had increased probabilities of having ≥3 times a grade of 8 or higher in five clerkships. Research question 2: when supervisors were allowed to deviate from original grades, ethnicity‐related differences in clinical grades were reintroduced.

**Conclusions:**

Broadly sampled assessment reduces ethnicity‐related differences in grades.

## Abstract 2

van Andel et al. demonstrate that ethnicity‐related differences in clinical grades can be reduced using multiple assessors, multiple assessments and multiple evaluation moments.

## Introduction

Ethnic majority students achieve higher grades compared with ethnic minority students in the pre‐clinical^1^, ^2^ and in the clinical training phase,^3^ across different types of written and clinical examinations.^4^ This discrepancy favouring ethnic majority students is unexplained by previous medical school performance^3^, ^5^ and may result in long‐term benefits and increased career chances for ethnic majority students, which is undesirable given the societal benefits of a diverse medical workforce.^6^

Potential explanations for ethnic disparities in grades can be related to students themselves,^7^, ^8^ such as students’ time spent on homework,^9^ self‐efficacy,^10^ social network^8^ and course‐related enjoyment.^11^ Explanations can also be found in educational learning environments,^12^ which include assessors and assessments. For instance, assessors may develop stereotypical expectations of students, and these could influence students’ evaluations.^13^ Assessors’ subjectivity in clinical grades has been well described in the assessment literature and reflects variance in evaluations between assessors.^14^, ^15^, ^16^, ^17^ Some medical education researchers argue that assessors’ subjectivity can be reduced, others argue that it cannot be avoided, and again others argue that subjectivity is meaningful and comes from expert judgements that have legitimate experience‐based interpretations.^18^ Evaluation of students’ competencies with mixed types of assessments used by multiple assessors across multiple moments (referring to a broad sampling method) has been suggested to maximise expert judgements and minimise unwarranted variances in evaluations.^18^, ^19^, ^20^, ^21^ However, it has not been investigated whether broadly sampled assessment could function to reduce ethnic disparities in clinical performance evaluations.

A broadly sampled assessment implies that multiple assessors obtain information from various assessment sources, after which all assessment sources are aggregated to make a richly informed grading decision. By contrast, a global performance rating implies that an assessor integrates judgements about competencies into one overall score. Presumably, the optimal type of assessment depends on the type of competency assessed.^22^ Clinical knowledge might be best evaluated by machines,^20^ such as the multiple‐choice examination,^23^ yet most other clinical competencies (such as communication or collaboration) are arguably socially determined^14^ and complex,^24^ and might therefore be best evaluated by human assessors. When it is difficult to obtain a representative evaluation, a sample of mixed assessments may be useful. Researchers who conduct case studies in educational settings, for instance, often use mixed methods because they want to both generalise their findings and have an in‐depth understanding of the context.^25^

Subjective ratings of individual abilities can form reliable and valid measures,^26^, ^27^, ^28^, ^29^ but researchers often describe such ratings as problematic in the absence of clearly articulated standards,^30^ and when observations occur too infrequently.^19^, ^21^ A broad sampling technique could give a more generalisable indication of students’ competencies.^31^ Such a technique could partially compensate for random variance in evaluations that are not related to the true competencies of students, such as chance and situational factors.^16^, ^21^ In other words, for students, some patient cases can be more difficult than others, and likewise, some assessors can be more stringent than others, and this affects their evaluation. Efforts to reduce irrelevant variability are desirable, given that assessors are likely to be influenced as much by irrelevant students’ characteristics (e.g. skin colour, gender and accent) as they are by the content of students’ performance.^17^ Assessors have a tendency to categorise medical students according to personality inferences and behavioural interpretations, even if these are not related to competencies.^32^, ^33^ If broad sampling has the potential to reduce the effects of irrelevant variances (i.e. variances that are not related to students’ competencies themselves),^16^, ^21^ then such an assessment method might also reduce the effect of variance related to ethnicity. Therefore, it is expected that a broadly sampled assessment, as opposed to a global assessment, mitigates ethnic disparities in clinical performance evaluations.

Final grade decisions in broadly sampled assessments are determined by an aggregation of numerous data points from various rich information sources. A challenge remains regarding how these various data points should be integrated. Using a formula or algorithm for final evaluations, rather than human judgement alone, is often recommended because a formula or algorithm has been shown to produce more accurate, reliable and consistent predictions.^34^, ^35^ This is partly because assessors can be biased in their recall of what has occurred in clinical evaluations.^18^ Assessors might, for instance, subconsciously form judgements (and ‘fill in the blanks’) based on stereotype‐consistent information.^36^, ^37^ Hence, it is expected that ethnicity‐related differences in clinical grades are more likely to occur when supervisors are given the opportunity to determine a final grade with their own judgements, than when an algorithm is used as a guide.

**Figure 1:**
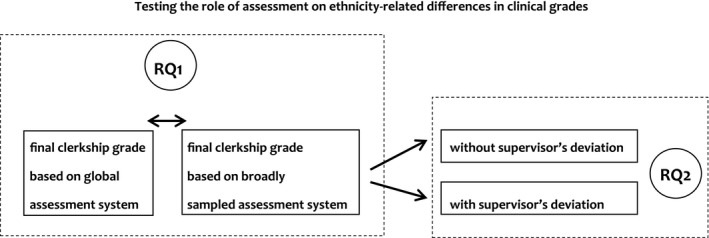
Schematic representation of the two research questions. Research question 1 (RQ1): tests the effect of two different assessment systems on ethnicity‐related differences in clinical grades. Research question 2 (RQ2): tests the effect of whether or not supervisors had deviated from original broadly sampled scores on ethnicity‐related differences in clinical grades

Our medical school recently moved from a global to a broadly sampled assessment for clinical evaluations, including a final grade that is based on an algorithm. However, supervisors are allowed to deviate by one full grade point. This development enabled us to conduct a study that addresses two research questions: (RQ1) Do ethnicity‐related differences in clinical grades decrease when assessed in a broadly sampled assessment as compared with a global assessment?, and (RQ2) ethnicity‐related differences in clinical grades increase when supervisors are given the opportunity to deviate from the broadly sampled assessment score (i.e. from an algorithm)? (See Fig. 1 for a schematic representation of our research questions.)

## Methods

### Context

This study is a retrospective cohort study and was conducted at the Erasmus MC Medical School in Rotterdam, the Netherlands. This school has a relatively large number (~30%) of ethnic minority students compared with other Dutch medical schools. The master phase of the medical course covers 3 years. It consists of thematic education and master research in the first year, and 12 discipline‐specific clerkships in the second and third years. Clerkships take place in a fixed sequence and include the following: internal medicine (10 weeks), surgery (10 weeks), paediatrics (5 weeks), psychiatry (5 weeks), neurology (5 weeks), gynaecology (5 weeks), dermatology (3 weeks), ear, nose and throat surgery (3 weeks), ophthalmology (3 weeks), general practice (5 weeks), social medicine (2 weeks) and rehabilitation (1 week).

### Global versus broadly sampled assessment

Before 2012, using global assessment, the evaluation of a student's performance during the master phase consisted of a global performance rating (GPR) per clerkship. This GPR represents a global rating awarded by a supervisor, covering a student's performance on six different clinically relevant competencies over the clerkship period.^38^ These competencies are identified and described by the CanMeds (Canadian Medical Education Directives for Specialists) framework and include the following: (i) medical expert, (ii) scholar, (iii) communicator, (iv) health advocate, (v) collaborator, and (vi) organiser. Each student receives an overall GPR for these competencies at the end of each discipline‐specific clerkship, which is based on patient‐related and oral evaluations.

In order to make more accurate predictions of students’ competencies, a broadly sampled assessment was then implemented in 2012. This assessment examines the same competencies as the global assessment in terms of evaluation criteria, but differs in terms of evaluation procedure (see Table 1). Students in this system are still evaluated on the basis of the same six clinically relevant competencies, yet these competencies are formally evaluated in five different assessments by at least two assessors on multiple occasions. These five assessments include a knowledge‐based assessment, as measured by a computer, and four competency‐based assessments, as measured by assessors. The master knowledge test of internal medicine is the only test that is taken orally rather than by computer. Students still receive a final clerkship grade from their supervisor, but this grade results from an algorithm for partial grades (‘if three or more partial grades are above average then the final grade equals 8, and if one partial grade is below average then the final grade equals 6’, etc.).

**Table 1:** An illustration of how broadly sampled assessment takes place, and how it leads to a final clerkship grade

	Medical expert	Scholar	Communicator	Health advocate	Collaborator	Organiser	Final clerkship grade
Master Knowledge Test	x						
Observational patient contact 1	x		x	x			
Observational patient contact 2	x		x	x			
Daily functioning	x		x		x	x	
Reflection and feedback		x					
Final judgement per competency	x	x	x	x	x	x	x

Six medical competencies are evaluated in five different assessments, at multiple moments, by at least two assessors. Final judgements per competency form a final clerkship grade via an algorithm.

Hence, the broadly sampled assessment is similar to the global assessment in terms of which clinically relevant competencies are valued and graded, but differs in four important ways: (i) multiple evaluation moments, (ii) multiple assessors, (iii) assessments by both humans and the computer, and (iv) partial grades for specific competencies are made explicit and integrated by an algorithm into a final grade. Although the final clerkship grade is primarily computed by an algorithm, a student's supervisor is allowed to deviate from this computed clerkship grade by a maximum of one full grade point (on a scale from 1 = poor, to 10 = excellent).

### Participants and procedure

The present study included 1667 students (65.5% female) and consisted of students who had completed their first five clerkships in the cohorts between 2002 and 2004 (*n = *818 for global assessment), and students who had completed their first five clerkships in the cohorts between 2008 and 2010 (*n *=* *849 for broadly sampled assessment). A total of 10 students were excluded from analysis because they belonged to cohorts that had been globally assessed, but were eventually assessed with broad samples. Cohorts refer to the years when medical students entered medical school. These cohorts were selected for comparability reasons, as both samples have similar sample sizes and include three cohorts. Also, this selection of cohorts prevented overlap; the excluded cohorts between 2005 and 2007 included students from both assessments. Grades on the first five clerkships that students followed were chosen because this enabled data collection for the most recent cohorts. Grades on the first five clerkships have been shown to be a good representation of grades for all 10 clerkships.^39^ Note that failure to complete clinical training is rare (approximately 1% in this medical school).

### Ethical approval

Data on ethnicity, (im)migration status, gender and age (at the moment students entered medical school) for these cohorts were available from a national database of students in higher education in the Netherlands, which is called 1CijferHO. The Dutch Data Protection Authority contributed to and approved data collection for our study. Evaluation scores, including average bachelor grade, were derived from the university student administration system and confidentiality was guaranteed. As grades were collected as part of regular academic activities, individual consent was not necessary.

### Variables and measures

#### Student characteristics

According to Statistics CBS (www.CBS.nl, the Netherlands), an individual belongs to an ethnic minority group if at least one of his or her parents was born outside of the Netherlands. Based on the country of birth of students’ parents, students were classified into one of five ‘ethnic student groups’: native Dutch; Turkish/Moroccan/African; Surinamese/Antillean; Asian, and Western. Surinamese/Antillean ethnic student groups included students with a migration background in Dutch Guyana. The Asian ethnic student group mainly included China, and Afghanistan, Iraq, Iran and Pakistan. The Western ethnic student group included all countries in Europe (except for the Netherlands) and North America, Oceania, Japan and Indonesia.

‘Age’ was categorised as ‘<19 years old’, ‘19–21 years old’, and ‘>21 years old’ at the moment students entered medical school. ‘First‐generation immigrants’ (*no/yes*) referred to whether or not ethnic minority students were born outside the Netherlands. ‘Average bachelor grade’ was recorded as a mean grade on a 10‐point scale after completion of the initial 3 years of medical school (1 = very poor, 10 = excellent). Students who were evaluated by the global assessment received an average bachelor grade for their second, third and fourth year of medicine, rather than the first 3 years, because these students were officially graduated with a doctorate degree rather than a bachelor degree. Further, ‘assessment’ (global versus broadly sampled) referred to whether students were broadly sampled or globally assessed.

#### Dependent measures

*‘*Good clinical evaluation*’* was defined as achieving an 8 or higher in at least three out of five clerkships *(yes/no)*. The first five clerkships included internal medicine, surgery, paediatrics, neurology and psychiatry. The achievement of an above‐average grade more than half of the time (at least three out of five grades) can be seen as a representation of good clinical evaluation, and it increases the chances of being selected for a medical specialty residency of choice.^40^ Further, the ‘sum of assessors’ deviations’ was defined as the total of positive and negative grade point deviations a student received from supervisors in the first five clerkships. However, internal medicine was excluded from the analysis as this clerkship used a different algorithm to the other clerkships. The sum of deviations could therefore range from −4 (negative deviation) to +4 (positive deviation). An individual supervisor could upgrade (+1 grade point) or downgrade (−1 grade point) the original broadly sampled assessment score at the end of four clerkships.

### Statistical analysis

For RQ1, binary logistic regression odds ratios (ORs) were estimated in order to test whether student ethnicity predicts good clinical evaluation. Statistical interaction terms expressed potentially differential effects of assessment on the relationship between students’ ethnicity and clinical evaluation. A 95 percent confidence interval was displayed for unadjusted and adjusted ORs (adjusted ORs implied that these were controlled for students’ gender, age, average bachelor grade and first‐generation immigration status). Statistical significance indicates that OR values do not include 1.0. For RQ2, linear regression analyses were performed, first without and then with adjustments, in order to test the effect of student ethnicity on receiving upgrades from supervisors. Analyses were performed using IBM spss Statistics Data Editor, Version 22.0 (IBM Corp., Armonk, NY, USA).

## Results

### Student characteristics of both samples

The student samples from both assessments had the same percentage of female students (65.5%) and did not statistically differ with regard to age (*χ* ^2^ (1, *n *=* *1667) = 2.89, p* *=* *0.24), ethnicity (*χ* ^2^ (1, *n *=* *1667) = 2.89, p* *=* *0.24), first‐generation immigration status (*χ* ^2^ (1, *n* = 1667) = 0.02, p* *=* *0.89) or average bachelor grade (*F* (1, *n* = 1666) = 2.67, p* *=* *0.10). For students’ characteristics across ethnic student groups per assessment, see Table 2.

**Table 2:** Descriptive statistics across ethnic student groups per assessment

	Dutch		T/M/A		S/A		Asian		Western		p
	*n*	%	*n*	%	*n*	%	*n*	%	*n*	%	
	623	76.2	37	4.5	37	4.5	40	4.9	81	9.9	
Global assessment											
Gender (female)	416	66.8	24	64.9	25	67.6	17	42.5	54	66.7	0.04
Age <19 years	389	62.4	17	45.9	19	51.4	12	30.0	40	49.4	
Age 19–21 years	169	27.1	14	37.8	14	37.8	13	32.5	33	40.7	
Age >21 years	65	10.4	6	16.2	4	10.8	15	37.5	8	9.9	0.00
First‐generation immigrant (yes)	0	0.0	7	18.9	15	40.5	29	72.5	20	24.7	0.00
≥3 times grade 8 or higher	545	87.5	25	67.6	20	54.1	23	57.5	62	76.5	0.00
	**Mean**	**SD**	**Mean**	**SD**	**Mean**	**SD**	**Mean**	**SD**	**Mean**	**SD**	
Average bachelor grade	6.65	0.46	6.52	0.42	6.49	.42	6.46	.46	6.64	.47	0.01
Average clinical performance	7.94	0.38	7.80	0.48	7.60	0.41	7.64	0.52	7.86	0.41	0.00

	Dutch		T/M/A		S/A		Asian		Western		p
	*n*	%	*n*	%	*n*	%	*n*	%	*n*	%	
	615	72.4	54	6.4	46	5.4	57	6.7	77	9.1	
Broadly sampled assessment											
Gender (female)	414	67.3	31	57.4	32	69.6	34	59.6	45	58.4	0.25
Age <19 years	359	58.4	27	50.0	22	47.8	18	31.6	36	46.8	
Age 19–21 years	188	30.6	21	38.9	18	39.1	29	50.9	27	35.1	
Age >21 years	68	11.1	6	11.1	6	13.0	10	17.5	14	18.2	0.01
First‐generation immigrant (yes)	0	0.0	4	7.4	16	34.8	32	56.1	20	26.0	0.00
≥3 times grade 8 or higher	402	65.4	27	50.0	28	60.9	27	47.4	48	62.3	0.03
	**Mean**	**SD**	**Mean**	**SD**	**Mean**	**SD**	**Mean**	**SD**	**Mean**	**SD**	
Average bachelor grade	6.70	0.45	6.48	0.32	6.52	0.35	6.60	0.44	6.66	0.41	0.00
Average clinical performance	7.70	0.41	7.52	0.48	7.62	0.37	7.48	0.51	7.69	0.47	0.00

T/M/A, Turkish/Moroccan/African; S/A,  Surinamese/Antillean; SD, standard deviation.

### RQ1: ethnicity‐related differences in grades in broadly sampled versus global assessment

**Figure 2:**
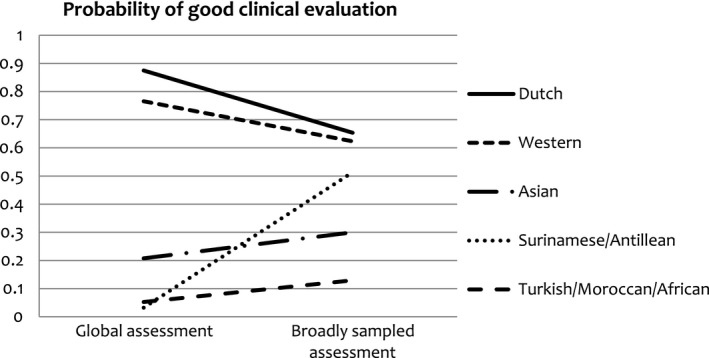
Probabilities for good clinical evaluation per assessment across ethnic student groups

A logistic regression model with students’ ethnicity, assessment, and the interaction of students’ ethnicity and assessment, predicting clinical evaluation (i.e. good clinical evaluation, defined as whether a student received at least three times an 8 or higher in five clerkships) showed that the overall model was significant (*χ* ^2^ (9) = 142.74, p<* *0.01). Assessment significantly predicted good clinical evaluation, implying that higher clinical evaluations were received in the global assessment (675 evaluations, 82.5%) as compared with the broadly sampled assessment (532 evaluations, 62.7%) (Wald *χ* ^2^ (1) = 78.45, p<* *0.01). Students’ ethnicity also significantly predicted good clinical evaluation (Wald *χ* ^2^ (4) = 49.95, p<* *0.01), favouring native Dutch students. Furthermore, the interaction between students’ ethnicity and type of assessment was significant (Wald *χ* ^2^ (4) = 15.77, p<* *0.01). The score differences between Surinamese/Antillean students and native Dutch students (Wald *χ* ^2^ (1) = 11.37, p<* *0.01) and between Asian students and native Dutch students (Wald *χ* ^2^ (1) = 4.18, p<* *0.05) were smaller in the broadly sampled assessment than in the global assessment. Turkish/Moroccan/African students and native Dutch students (Wald *χ* ^2^ (1) = 1.51, p* *=* *0.22) and Western students and native Dutch students (Wald *χ* ^2^ (1) = 2.72, p* *=* *0.10) showed non‐significant score differences when assessments were compared, even though the differences were in the predicted direction. The results showed that native Dutch students had significantly reduced chances, whereas Surinamese/Antillean and Asian students had significantly increased chances, of having a good clinical evaluation in broadly sampled assessment as compared with global assessment. Table 3 shows the unadjusted ORs per assessment across ethnic student groups and Fig. 2 displays the above interaction effect with probabilities for good clinical evaluations.

**Table 3:** The estimated odd ratios (95% confidence interval) of receiving an 8 or higher for at least three out of five clerkships across ethnic student groups per assessment

	Global assessment	Broadly sampled assessment
Unadjusted ORs for good clinical evaluation		
Dutch ethnicity	1	1
Turkish/Moroccan/African ethnicity	0.30 (0.14–0.62)**	0.53 (0.30–0.93)*
Surinamese/Antillean ethnicity	0.17 (0.08–0.34)**	0.82 (0.45–1.52)
Asian ethnicity	0.19 (0.10–0.38)**	0.48 (0.28–0.82)**
Western ethnicity	0.47 (0.27–0.82)**	0.88 (0.54–1.43)
Adjusted ORs for good clinical evaluation		
Dutch ethnicity	1	1
Turkish/Moroccan/African ethnicity	0.33 (0.15–0.70)**	0.71 (0.40–1.27)
Surinamese/Antillean ethnicity	0.17 (0.08–0.36)**	1.02 (0.52–2.01)
Asian ethnicity	0.22 (0.09–0.55)**	0.52 (0.26–1.01)
Western ethnicity	0.43 (0.23–0.79)**	0.94 (0.55–1.61)

Adjusted ORs are controlled for gender, age, average bachelor grade and first‐generation immigration status.

*p< 0.05 and **p< 0.01 compared with the Dutch reference group. OR, odds ratio.

The same analyses were conducted, but now adjusted for gender, age, first‐generation immigration status and average bachelor grade. The overall model again was significant (*χ* ^2^ (13) = 265.39, p<* *0.01). Students’ ethnicity, type of assessment, and the interaction between students’ ethnicity and type of assessment, remained predictors of good clinical evaluations. Being female (Wald *χ* ^2^ (1) = 12.81, p<* *0.01) and having a high average bachelor grade (Wald *χ* ^2^ (1) = 1.58, p<* *0.01) both positively and independently influenced the chance of receiving a good clinical evaluation. Neither age (Wald *χ* ^2^ (1) = 0.51, p* *=* *0.48) nor first‐generation immigration status (Wald *χ* ^2^ (1) = 0.43, p* *=* *0.51) had a significant effect on a good clinical evaluation. When analyses were performed including adjustments, the data showed significant ethnicity‐related differences in clinical grades in global assessment, but not in broadly sampled assessment (see Table 3). The adjusted ORs in global assessment correspond to Cohen's small to medium effect sizes.^41^

### RQ2: ethnicity‐related differences before and after supervisors’ deviations from the original broadly sampled assessment score

The broadly sampled assessment included grades from 849 students (65.5% female, see Table 2 for student characteristics). The clerkships related to psychiatry and surgery showed the highest relative number of upgrades, whereas neurology and paediatrics showed the highest relative number of downgrades (see Table 4). Upgrading happened for approximately half of the cases (between 48.9% and 55.7%), whereas downgrading was rare (between 1.3% and 2.9%).

**Table 4:** Overall frequencies and proportions of student cases for whom broadly sampled test scores remained the same, and for whom downgrades and upgrades were given

	Downgrade (−1)		Remains broadly sampled score		Upgrade (+1)	
	*n*	%	*n*	%	*n*	%
Surgery	11	1.3	376	44.3	462	54.4
Paediatrics	25	2.9	473	55.7	351	41.3
Psychiatry	16	1.9	415	48.9	418	49.2
Neurology	24	2.8	460	54.2	365	43.0

Linear regression analysis showed that student ethnicity had a main effect on the total number of upgrades (*F*(4) = 2.38, p* *=* *0.05). Asian students (β = −0.33, p* *=* *0.06 [marginally significant]) and Turkish/Moroccan/African students (β = −0.43, p* *=* *0.02) were less likely to receive upgrades, as compared with native Dutch students. These effects were no longer significant after controlling for gender, age, first‐generation immigration status and average bachelor grade. Additional linear regression analyses showed that average bachelor grade accounted for substantial variance (medium effect size^42^) in the total number of upgrades (β = 0.79, p<* *0.01). Furthermore, the results showed that average bachelor grade itself was also related to students’ ethnicity. Both Surinamese/Antillean students (β = −0.19, p<* *0.01) and Turkish/Moroccan/African students (β = −0.22, p<* *0.01) scored lower than native Dutch students in their average bachelor grades. The differences between native Dutch students, Asian students (β = −0.10, p* *=* *0.11) and Western students (β = −0.04, p* *=* *0.42) were non‐significant.

## Discussion

First, the findings showed that a broadly sampled assessment, which involves mixed types of assessment used by multiple assessors across multiple occasions, decreases ethnicity‐related differences in clinical grades. Native Dutch students have significantly reduced chances, whereas other non‐Western ethnic minority students have significantly increased chances, of having good clinical evaluations in broadly sampled as compared with global assessment. This result was visible even after adjustments. Second, the findings showed that when supervisors are given the opportunity to deviate from the suggested or computed broadly sampled assessment score, ethnicity‐related differences in clinical grades are re‐introduced. Final grade decisions are the uncontaminated result of an algorithm for only half of the cases, implying that supervisors deviate from original grades in the other half of the cases. Native Dutch students, as compared with other ethnic student groups, are then more likely to receive positive deviations (that is, one grade point higher) from their supervisors at the end of their clerkships. Average bachelor grade, a variable that was partly dependent on students’ ethnicity, also predicted the total number of positive deviations.

Overall, the first results showed that clinical evaluations of students with different ethnicities become more similar in broadly sampled assessment as compared with global assessment. A possible explanation is that broad sampling extends the degree to which scores generalise to the domain of interest, and therefore, more reliable estimates can be obtained.^43^ Consequently, broad sampling reduces the effect of random error or variance amongst groups,^16^, ^21^ including student groups who differ based on ethnicity. It is critical to understand that broad sampling does not fully reduce ethnic bias, which is a systematic error, as this evaluation system might still contain assessors who are prejudiced or biased. Only adding multiple evaluation moments might to some degree decrease this type of bias, as more encounters provide more opportunity to receive information that is inconsistent with stereotypical expectations (i.e. ‘forecasting error’).^44^

A second finding indicated that using an algorithm for broadly sampled assessments is arguably more preferred, because when ethnic majority supervisors are allowed to deviate from algorithm scores, they tend to favour ethnic majority students, relative to ethnic minority students. An explanation can be found in previous research that has shown how assessors are inclined to recall people according to stereotype‐consistent judgements.^18^, ^36^, ^37^ Psychological research has consistently shown that ethnic majorities, as compared with ethnic minorities, are more likely to be positively evaluated because they belong to the assessors’ in‐group and share similarities with the ethnic majority evaluator.^45^ Research on intergroup discrimination in cooperative decision making has shown that in‐group favouritism, which refers to a more positive evaluation of in‐groups as compared with out‐groups, is more likely to occur than out‐group derogation, which refers to a more negative evaluation of out‐groups as compared with in‐groups. This might suggest that standardisation by implementing an algorithm for grade decisions mitigates in‐group favouritism towards ethnic majority students. Algorithms probably provide more accurate, reliable and consistent predictions.^34^, ^35^ Also, when assessors are held accountable, by asking them to legitimate their evaluation decisions, grade differences as a result of ethnicity (and other irrelevant information) can be reduced. Indeed, research has shown that when decision makers are held accountable for making fair selections, qualifications of candidates play a more vital role and their biases tend to reduce.^46^, ^47^

The study's findings are in line with our expectations and earlier research, except for the finding that average bachelor grade was partly able to explain the relationship between students’ ethnicity and receiving an upgrade. However, our data showed that average bachelor grade itself was also influenced by students’ ethnicity. It might therefore have been the case that ethnic minority students made fewer displays of their clinical knowledge, because of lower pre‐clinical evaluations, and that supervisors were therefore less inclined to give upgrades. Clinical knowledge, rather than other competencies, is mainly acquired and tested during the pre‐clinical phase and is heavily weighted in the role of medical expertise according to the CanMEDS framework. It may therefore be speculated that displays of clinical knowledge have partly resulted in higher evaluations.

Broad sampling in assessment^18^, ^19^, ^20^, ^21^ could compensate for variance in evaluations that is not a result of true differences in competencies amongst students,^16^ and this might perhaps be a result of more structure. With broad sampling, supervisors explicitly need to give partial grades per competence (e.g. the roles of medical expert, communicator and health advocate need to be evaluated explicitly in the assessment of observational patient contact; see Table 1). Structure in evaluations has been shown to reduce irrelevant variance as an input for evaluations in employment interviews,^48^ and more generally it has been shown to decrease group differences that are a result of gender and race.^49^, ^50^ Future research could, therefore, more closely examine the accompanying effect of structure in broad sampling.

Our study has a few limitations and strengths. Broadly sampled assessment has been argued to improve the validity and reliability of evaluations. However, predictive validity and reliability measurements were beyond the scope of this study and could not have been compared because global assessments only use one overall score. It would be interesting to measure how medical students are performing as doctors, to estimate the predictive validity of broadly sampled assessment. Another limitation is that the ethnicities of assessors were not taken into account, which might have played a role in their evaluations. Those ethnicities were untracked, although we know that the majority of assessors in our medical school are native Dutch. Future research needs to investigate the effect of assessors’ ethnicity on the relationship between students’ ethnicity and clinical evaluations. Further, as differences in grades are likely to be caused by many factors,^51^ one could state that the evaluation differences found are not because of differences in assessment method, but that they are rather related to students themselves, or changes in faculty development. A strength of our study, however, is that these two student samples were similar with regard to basic student characteristics (gender, age, average bachelor grade and immigration status). Also, there were no reasons to expect that some curriculum changes, such as slight changes of course content, would differentially affect students with different ethnic backgrounds. Another strength of our study is that we were able to include large sample sizes (~800 students), and approximately a third of the participants were ethnic minority students.

With regard to the logical challenges and practical implications of moving to a broadly sampled assessment of clinical competencies, it was a challenge to schedule multiple evaluations at the end of the clerkships, in particular for the shorter clerkships (≤ 3 weeks). Another challenge was the tension between increasing standardisation by implementing an algorithm and also maintaining assessors’ freedom. Initially, assessors had difficulties with accepting the original scores that followed from the broadly sampled assessments, which was the reason for allowing them to deviate by one full grade point.

The development of a good assessment system to measure clinical competencies has been a challenge in medical education due to the shift towards competency‐based education.^52^ Assessors’ subjectivity in clinical grades has been widely recognised, yet interventions, such as so‐called anti‐prejudice messages^53^ or concentrated cognitive retraining,^54^ have been demonstrated to be unsuccessful in the reduction of stereotype application. Our study recommends that policymakers use multiple sources of information from various assessment methods and assessors to form clinical evaluations. Broad sampling can compensate for assessment or assessor flaws and allows patterns in students’ competencies to emerge.^55^ It is not recommended to enable supervisors to deviate from broadly sampled scores, especially when they are not being held accountable. An algorithm can integrate and aggregate numerous data points into valid and reliable predictions, including when individual data points are subjective ratings from experts.

This retrospective cohort study was explorative in nature and we invite other researchers to (dis)confirm our findings in replication research. This study shows that a broadly sampled assessment is able to reduce ethnic disparities in clinical evaluations and recommends aggregating data into final grades on the basis of an algorithm, rather than judgement by a clinician. In sum, broadly sampled assessment seems to contribute to a more diverse and inclusive educational environment.

## Contributors

All authors (CvA, KS‐J, AT and MB) were involved in the conception and design of the study. KS‐J collected the data and CvA analysed the data. All authors contributed to the interpretation of the data. CvA wrote the first draft of the research paper. All authors contributed to the critical revision of the paper and approved the final manuscript for publication. All authors are accountable for the manuscript.

## Funding

no funding was received for this study.

## Ethical approval

this research was carried out in accordance with the Declaration of Helsinki. The Dutch Data Protection Authority contributed to and approved data collection. Individual consent was not necessary, given that the collection of clinical grades was part of regular academic activities.
